# Herniation of the Anterior Wall of the Stomach into a Congenital Postdiaphragmatic Space: An Unusual Complication following Laparoscopic Nissen Fundoplication

**DOI:** 10.1155/2010/893017

**Published:** 2010-11-22

**Authors:** A. Eisawi, A. Al-Temimi, Mohamed Dirie, S. Bukhari, M. Siddiqui

**Affiliations:** Department of General Surgery, Queen Elizabeth Hospital, London SE18 4QH, UK

## Abstract

Postoperative herniation of the stomach into potential spaces is a rare but serious complication of Nissen fundoplication. We report a 55-year-old female who presented with persistent vomiting shortly following laparoscopic Nissen fundoplication. At laparotomy, the anterior wall of the stomach was noted to be herniating into a congenital space behind the diaphragm. Anterior gastropexy was performed following the reduction of the herniating gastric segment. A high index of suspicion followed by aggressive and timely intervention is necessary to diagnose and manage postoperative gastric herniation and reduce the subsequent morbidity and mortality.

## 1. Introduction

The advent of laparoscopic surgery has revolutionised the surgical treatment of gastro-oesophageal reflux disease (GORD) resulting in a significant increase in the number of operations performed.Studies have consistently shown that the functional results of laparoscopic antireflux procedures are equal to open surgery but with significantly less postoperative morbidity and a shorter hospital stay [1–3].

The surgical management of GORD sometimes fails,whether performed as an open procedure or laparoscopically, and may require a reoperation for optimal results [4]. Amongst the various mechanisms of failure, migration of the stomach into potential spaces such as in paraoesophageal herniation is a serious and important complication as the diagnosis may be significantly delayed resulting in an increase in morbidity and mortality of the affected patients [5–7]. In this report, we describe a case of migration of the stomach into a congenital postdiaphragmatic space following laparoscopic Nissen Fundoplication.

## 2. Case Presentation

A 55-year-old lady underwent an elective laparoscopic Nissen fundoplication. On the second postoperative day she deteriorated with persistent vomiting, pyrexia, and low oxygen saturations and was promptly admitted to the intensive care unit (ICU). Oesophago-gastro-duodenoscopy (OGD) was performed with dilatation of a tight gastro-oesophageal junction (GOJ).

She then continued to be hypoxic and hypotensive and a chest X-ray revealed a right sided pneumothorax which was managed with a right chest drain. Serial arterial blood gas samples did not reveal any acid-base disturbances at this stage. A gastrografin swallow ruled out any perforation in the oesophagus and was otherwise nonspecific. After a short period of noninvasive ventilation, she remained hypoxic and was consequently intubated. A second chest drain was inserted on the left side due to worsening bilateral pleural effusions on a subsequent chest X-ray.

**Figure 1:**
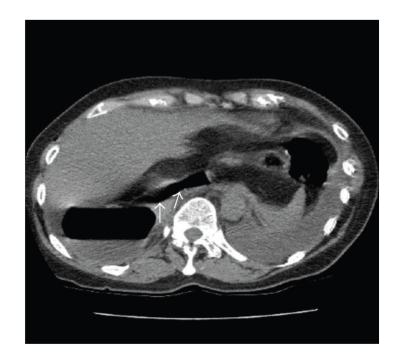
Computerised Tomography (CT) scan herniation of the stomach (Arrows-gastric air within the stomach) into a congenital post-diaphragmatic space.

A repeat OGD revealed a normal GOJ with a rotated and distended stomach but there was no evidence of gastric outlet obstruction. As the patient continued to deteriorate, a computerised tomography (CT) scan was done (Figure 1) which confirmed herniation of the stomach into a congenital postdiaphragmatic space. At exploratory laparotomy, the anterior wall of the stomach was noted to be herniating into a congenital space behind the diaphragm pushing the liver and the inferior vena cava anteriorly. There was no evidence of a gastric volvulus. Following the retrieval of the herniating segment, the wrap was noted to be congested with an area of ischaemia which recovered promptly following reduction and no resection was necessary. The wrap was undone revealing a defect in the anterior wall of the stomach. The margins of the defect, which were of doubtful viability, were trimmed and the defect was closed with nonabsorbable sutures and covered with omentum. The stomach was then fixed to the anterior abdominal wall with nonabsorbable sutures. No defect was noted in the diaphragm where three hiatal interrupted nonabsorbable sutures, which were placed during the previous fundoplication and were noted with a normal length of abdominal oesophagus.

The patient made a swift recovery and was discharged from the ICU. Both drains were eventually removed prior to her discharge from the hospital on the seventh postoperative day. The patient remained asymptomatic with no reflux symptoms after a period of 2 years of followup leading to her discharge from our care.

## 3. Discussion

Although migration of Nissen fundoplication wraps has been previously described [5–7], there are no reports of migration into congenital spaces including congenital post diaphragmatic spaces in the literature. It is timely to raise awareness of this potentially serious complication and carefully present the associated clinical features [2, 3]. In our case, it is possible that the creation of pneumoperitoneum resulted in a residual pocket of gas within the space exposing it as a potential herniating space postoperatively. A further possibility is that a tight GOJ after fundoplication might have induced acute gastric dilatation and herniation. It is notable that the patient did not have specific symptoms apart from reflux to account for the presence of this congenital space.

Clinical features following complicated fundoplication procedures are variable and often nonspecific. In addition, some patients are asymptomatic. Such complications are usually described in association with the commoner intrathoracic herniation. The majority of patients presenting with a complicated fundoplication complain of recurrent heartburn, bloatedness, and/or dysphagia [2, 3]. Less frequent complaints include persistent nausea and vomiting, diarrhea, bloatedness, shortness of breath, and shoulder or chest pain. While asymptomatic patients may be managed expectantly, symptomatic patients should be managed with a high index of suspicion implementing serial physical examinations, diagnostic imaging such as CT scanning, and timely surgical intervention [3].

As described earlier, our patient presented with nonspecific clinical signs including hypoxia, hypotension, pyrexia, vomiting, a unilateral pneumothorax, and bilateral pleural effusions. Some of her nonspecific symptoms could be explained in the context of systemic inflammatory response syndrome. Worsening of her clinical condition despite aggressive conservative treatment in ICU was a prompt for further investigations and subsequent surgical intervention. Anterior gastropexy was considered to be the most appropriate surgical intervention in the context of the life-threatening clinical presentation. The retention of an adequate length of abdominal oesophagus with an intact oesophageal hiatus repaired by three nonabsorbable sutures could explain the resolution of reflux symptoms after a long period of followup.

##  Consent

Consent was obtained from patient.

##  Conflict of Interest

The authors declare that they have no conflict of interests.
